# The coevolution of recognition and social behavior

**DOI:** 10.1038/srep25813

**Published:** 2016-05-26

**Authors:** Rory Smead, Patrick Forber

**Affiliations:** 1 Department of Philosophy and Religion, Northeastern University, Holmes Hall, 360 Huntington Ave, Boston, MA 02115, USA; 2 Department of Philosophy, Tufts University, Miner Hall, 14 Upper Campus Rd, Medford, MA 02155, USA.

## Abstract

Recognition of behavioral types can facilitate the evolution of cooperation by enabling altruistic behavior to be directed at other cooperators and withheld from defectors. While much is known about the tendency for recognition to promote cooperation, relatively little is known about whether such a capacity can coevolve with the social behavior it supports. Here we use evolutionary game theory and multi-population dynamics to model the coevolution of social behavior and recognition. We show that conditional harming behavior enables the evolution and stability of social recognition, whereas conditional helping leads to a deterioration of recognition ability. Expanding the model to include a complex game where both helping and harming interactions are possible, we find that conditional harming behavior can stabilize recognition, and thereby lead to the evolution of conditional helping. Our model identifies a novel hypothesis for the evolution of cooperation: conditional harm may have coevolved with recognition first, thereby helping to establish the mechanisms necessary for the evolution of cooperation.

Cooperative behavior presents an evolutionary puzzle due to the destabilizing effects of defection or free-riding. Solutions to this puzzle involve ways of preferentially cooperating with other cooperative individuals or policing and punishing defection^1^,^2^,^3^,^4^,^5^,^6^,^7^,^8^,^9^,^10^,^11^,^12^. Many mechanisms for the evolution of cooperation presume some sort of capacity to recognize behavioral types, kin, or specific individuals. Coevolutionary models of recognition and social behavior have the potential to reveal new dynamical scenarios^13^,^14^,^15^. Here we show that if recognition coevolves with conditional strategies, costly conditional harming behavior can facilitate the evolution and stability of recognition whereas conditional helping behavior cannot. We model evolution using the discrete time replicator dynamics in a combined game (also called a Bayesian game) where both help and harm interactions occur with some probability^16^,^17^,^18^,^19^,^20^. We find that recognition tends to deteriorate in cooperative contexts. Once recognition deteriorates, cooperative behavior is easily destabilized. In contrast, conditional harming can coevolve with and maintain a high degree of recognition, potentially stabilizing both harming and helping behavior. Thus, while recognition can help cooperation evolve, the fitness benefits from cooperative interactions cannot solely explain the evolution or stability of recognition. Our results also raise questions about the prevailing account of how punishment evolves. Cooperation is presumed to evolve first among kin or small groups, often involving mutualistic interactions, then the scope of cooperative behavior expands with punishment helping to mitigate the increased risk of defection^3^,^8^,^21^,^22^,^23^. Our model reveals an alternative scenario: conditional harming evolves and promotes the evolution of recognition, thereby enabling cooperation to evolve later. Finally, there is a clear connection to evolutionary models of spite, a sort of costly harming behavior^12^,^24^,^25^,^26^. Taken together with our results, these models show how spite can initially evolve and pave the way for future cooperation.

In general, prosocial behavior (e.g., altruism) needs some degree of positive assortment to evolve, whereas antisocial behavior (e.g., spite) needs some degree of negative assortment^11^,^12^,^24^. Recognition can facilitate the evolution of either social behavior by generating the necessary assortment among behavioral types. For instance, the greenbeard gene in *Solenopsis invicta* enables worker ants to recognize the genotype of young reproductives, and these workers execute those queens that do not have their same genotype^27^. Without the ability to recognize similar individuals, we would not generally expect unconditional harm to evolve as it would be applied to all offspring indiscriminantly. Recognition can take a variety of forms, from identifying particular markers associated with behavioral types, as in the greenbeard case^15^,^28^, to keeping track of particular individuals and their past behaviors^2^ or their reputation^9^. While the importance of recognition in the evolution of social behavior is clear, how that recognition evolves is less so. Here we use evolutionary game theory to model how recognition ability of signals, signs or cues coevolves with behavioral types when both helping and harming interactions are possible.

## The Evolution of Recognition

**Figure 1:**
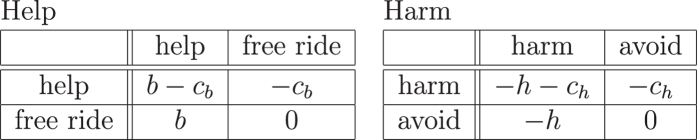
Normal forms for Help (*b* > *c*_*b*_ > 0) and Harm (h > *c*_*h*_ > 0) games.

Before presenting the complete model, it is helpful to see how recognition may evolve in simple contexts. Suppose that individuals of an infinite randomly mixing population may engage in either helping or harming behavior. We model each interaction as a distinct game (Fig. 1). The Help game has two strategies: an altruistic one (help) and an egoistic one (free ride). The Harm game also has two strategies: a spiteful one (harm) and an egoistic one (avoid). The altruistic strategy in the Help game involves an actor paying a cost *c*_*b*_ to confer a benefit *b* on a recipient. (If *b* > *c*_*b*_ then the Help game is a Prisoner’s Dilemma). The spiteful strategy in the Harm game involves an actor paying a cost *c*_*h*_ to inflict a harm *h* on a recipient. The egoistic strategies in both games avoid paying the costs and have no effect on recipients. Both the altruistic and spiteful strategies are strongly dominated by the egoistic strategy in their respective games. However, if individuals can behave conditionally based on recognizing the behavioral type of their opponent then these dominated strategies can evolve under a broad range of conditions^29^,^30^.

First consider the Help game. If types can perfectly recognize one another, the conditional strategy of acting altruistically only when paired with another conditional altruist can both invade and be evolutionarily stable with respect to the purely egoistic type provided that *b* − *c*_*b*_ > 0. The reason is that only conditional altruists receive benefits from the help behavior. In the Harm game, the conditional strategy of acting spitefully when paired with egoists and avoiding when playing others of the same type can, with respect to purely egoistic types, invade in restrictive conditions and when it does it is evolutionarily stable. More precisely, if the frequency of conditional spite (*x*) is such that *x* > *c*_*h*_/(*h* + *c*_*h*_), conditional spite will evolve and be maintained. In these limiting cases recognition facilitates helping behavior more so than harming behavior.

Both of these cases assume perfect recognition. However, recognition is seldom perfect in the biological world‒environments are noisy and deception in social interaction is rampant. Suppose we represent variable recognition ability of individuals with the parameter *r*. In the context of each interaction individuals may attempt to discern whether their opponent is the same behavioral type. With probability *r*, individuals correctly identify the type of another individual as *similar* or *different*. With probability (1 − *r*), individuals incorrectly identify the other individual, treating a different type as similar or vice versa. When *r* = 1/2 individuals are equally likely to treat a similar type as different and different types as similar. In other words, at *r* = 1/2 conditional behaviors are employed at random and do not track behavioral types at all. When *r* > 1/2 individuals are achieving some success at recognition, but when *r* < 1/2 individuals are misidentifying behavioral types more often than not. If *r* = 1 then recognition is perfect, and if *r* = 0 recognition always fails.

To model the evolution of recognition, we consider the fitness effects of introducing alternative recognition abilities *r*′ into a population. First, consider the evolution of recognition in populations playing only Help. A conditional altruist helps individuals recognized as similar and otherwise free rides. If recognition is sufficiently accurate (*r* > *c*_*b*_/*b*, a relationship that parallels Hamilton’s rule), this type can invade and fix in the population. Suppose a mutant conditional altruist with different recognition ability *r*′ is introduced into a population of conditional altruists. Other conditional altruists will treat this mutant as similar and so it will receive all the benefits that the natives do. However, if *r*′ < *r*, the mutant will be more likely to avoid paying the cost *c*_*b*_ than the natives and will have a strict fitness advantage. Failures of recognition permit covert free-riding so there is selection against successful recognition when conditional altruism is at high frequencies in the population. Conditional altruism will be destabilized by a deterioration in recognition ability.

Conversely, a parallel argument shows that higher recognition ability will have a fitness advantage in the Harm game when conditional spite is common. The conditional spiteful type harms individuals recognized different and avoids harming those recognized as similar. In a monomorphic population of conditionally spiteful individuals, a mutant with a different recognition ability *r*′ will avoid paying the cost *c*_*h*_ whenever recognition *succeeds*. Because failures in recognition are costly in this population, the mutant will invade whenever *r*′ > *r*. The comparison suggests that conditional harming behavior tends to scaffold recognition whereas conditional helping behavior tends to erode it. These observations call for a more general model that allows us to explore the evolutionary dynamics between recognition ability and multiple simultaneous interactions.

## The Coevolutionary Model

To model the coevolutionary dynamics we combine the two games into a more complex Bayesian game where individuals meet randomly and engage in a Help interaction with probability *p* or a Harm interaction with probability (1 − *p*). We consider four behavioral types:

- *E* (for egoism): free ride or avoid in all interactions.
- *A* (for conditional altruism): help (in Help interactions) if they recognize their partner as a similar type, otherwise free ride or avoid.
- *S* (for conditional spite): harm (in Harm interactions) if they recognize their partner as a different type, otherwise free ride or avoid.
- *C* (for the combination of conditional behaviors): help (in Help interactions) if they recognize their partner as a similar type and harm (in Harm interactions) if they recognize their partner as a different type, otherwise free ride or avoid.

Type *E* employs the dominant strategy for both Help and Harm. Types *A, S* implement the two basic conditional strategies from Help and Harm respectively, but act as an egoist in the other game. We do not consider types that always Help or always Harm because they are dominated strategies regardless of recognition ability cannot be evolutionarily stable. Type *C* implements a combination strategy that has a conditional response in both Help and Harm interactions.

When individuals interact, each independently attempts to recognize the type of the other individual. As above, this succeeds with probability *r* and fails with probability (1 − *r*). When the recognition succeeds they treat the other individual according to their type. When recognition fails, they treat similar individuals as different and different individuals as similar. We assume recognition is mediated by some signal, sign, or cue regarding behavioral type (*E, A, S, C*). Any set of recognition relations (i.e., which types are recognized as similar or different) can be modeled, including asymmetries. We examine three salient kinds of type recognition: (i) *exclusive*, (ii) *discerning*, and (iii) *context inclusive*.

The baseline case is *exclusive* recognition where behavioral types treat only the identical type as similar in all interactions, and treat all other types as different. For an opponent’s behavioral type to be recognized as similar in the exclusive sense that individual must have the identical dispositions in both Help and Harm interactions (e.g., *A* types recognize *A* as the same and *C* as different, even in Help interactions).

*Discerning* recognition compares dispositions only in the class of interactions where the individual has a conditional response. *A* types base recognition on behavior in Help, *S* on Harm, and *C* on both Help and Harm. This introduces an asymmetry in recognition since *C* types are more discerning than *A* or *S* types. So, for instance, *A* recognizes *C* as similar due to *C*’s conditional helping behavior in Help but *C* recognizes *A* as different due to *A*’s avoiding behavior in Harm. In effect, the *C* type links the conditional responses of *A* and *S* types and treats this combination as a novel behavioral type.

*Context inclusive* recognition works by comparing the behavioral dispositions of the opponent within the current interaction. This sort of recognition is symmetric but context dependent. *A* and *C* treat each other as similar in Help interactions but as different in Harm interactions, and conversely for *S* and *C*.

Each approach to modeling recognition generates different fitness comparisons among the types in the game (see Methods). Despite these differences all three ways of modeling recognition produce qualitatively similar results. Although exclusive recognition is perhaps the most straightforward case, discerning and context inclusive cases provide more dynamically salient ways for recognition to function. Discerning recognition introduces asymmetries among type-interactions with interesting dynamical consequences, whereas context inclusive recognition helps show how *C* types might originate (see Discussion).

We modeled the coevolution of behavioral types and recognition ability in two population spaces: one space representing the possible distributions of behavioral types, and a second representing the possible distributions of recognition ability. A given population is located at a point in both spaces. We used a multi-population discrete-time replicator dynamic with mutation where evolution occurs in both population spaces each generation. This represents simultaneous change in behavioral types and recognition ability. Using two population spaces to model the coevolution of recognition and conditional behavior in a single target biological population enables us to avoid several limiting assumptions about the target system. Specifically, we can remain neutral regarding how traits are transmitted across generations (e.g., whether there is genetic linkage), correlations between the variability of each type of trait in the population, and the relative rates of evolutionary change. By abstracting away from specific details of the target population, this approach can produce more robust formal results that readily generalize to broad sets of interactions. Furthermore, the multi-population dynamic allows us to avoid placing artificial restrictions on the strategy set necessary to make a single population model tractable.

The fitness of behavioral types each generation is determined by assuming random interaction among members of the population given the current mean recognition ability of the population. The fitness of varying recognition ability is determined by comparing the current mean recognition value with perfect recognition given the distribution of behavioral types in the population. See Methods for the fitness functions and details on the evolutionary dynamics.

## Results

**Figure 2:**
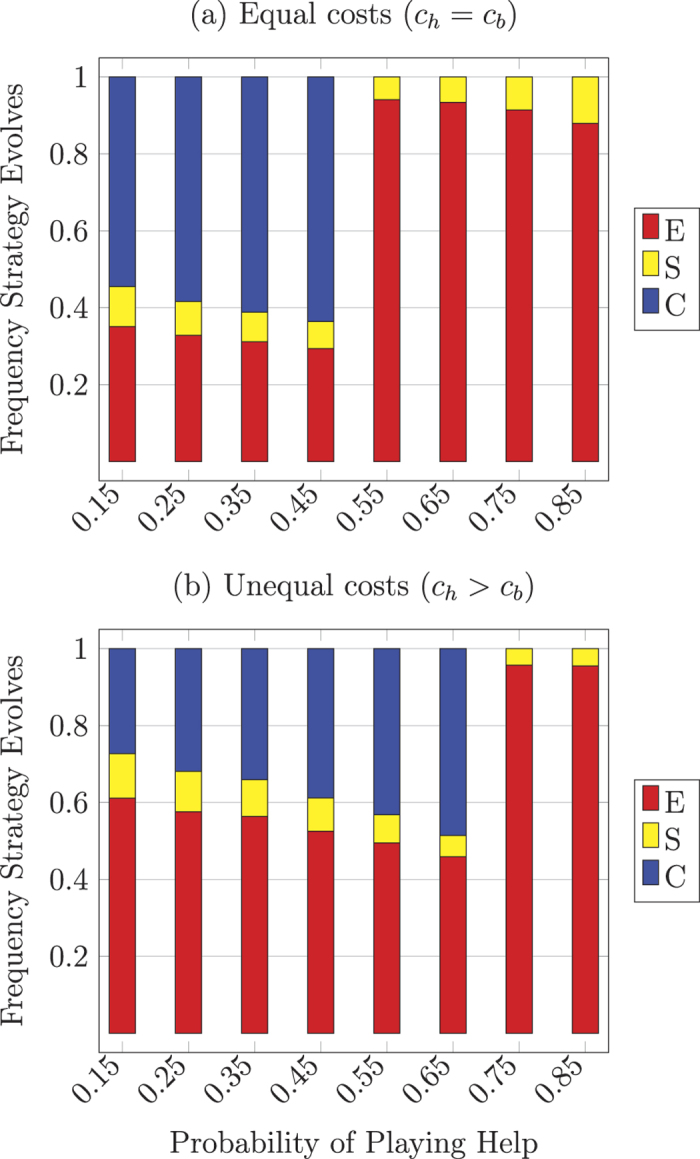
Simulation results for discerning (asymmetric) recognition: (**a**) with equal costs to confer help or inflict harm (*c*_*h*_ = *c*_*b*_ = 0.2) and (**b**) with a greater cost to inflict harm (*c*_*h*_ = 0.5, *c*_*b*_ = 0.2). All populations converge to a monomorphic equilibrium of one of the behavioral types *C, E*, or *S (A* never evolves). Simulation results show proportion of descendant population states from random initial conditions for equal values for help conferred or harm inflicted (*b* = *h* = 1), 10000 runs with mutation (*μ* = 0.0001).

We used numerical simulations to analyze the possible evolutionary outcomes and estimate the basins of attraction for the dynamically stable equilibria. Figure 2a summarizes the simulation results for discerning recognition across different values of *p* (the probability of engaging in Help interactions). Notice that the conditional helping type (*A*) always goes extinct, while the conditional harming type (*S*) can evolve in small range of conditions, usually those where *S* starts out at a relatively high initial frequency. The only conditional type that can reliably evolve across a wide range of initial conditions is *C*. However, *C* can only evolve in cases where Harm interactions are more probable than Help interactions. When *p* > 0.5 recognition *r* deteriorates and the egoist type *E* destabilizes *C*, but when *p* < 0.5 *C* is stable with respect to invasion by *E*. High recognition ability is maintained in *C* populations since the *C* type avoids harming each other in Harm interactions and effectively punishes invading types. When Harm interactions occur more frequently than Help interactions, the gains of free riding by recognition failure are offset by the added costs of additional spite. This promotes successful recognition and enables *C* to effectively eliminate other types.

The transition point for dynamical outcomes (from predominantly *E* to predominantly *C*) falls at *p* = 0.5 due to equal costs of helpful and harmful interactions in the the respective games: *c*_*b*_ = *c*_*h*_. More generally, the condition for the stability of *C* (with respect to invasion by *E*) is *h*/(*h* + *c*_*b*_ − *b*) ≥ *p* and the condition for the maintenance of *r* is *c*_*h*_/(*c*_*h*_ + *c*_*b*_) ≥ *p* (see Methods). Figure 2b summarizes simulation results for *c*_*b*_ ≠ *c*_*h*_. When *c*_*h*_ > *c*_*b*_ type *C* evolves at higher values of *p* but the basins of attraction at lower values of *p* decrease.

**Figure 3:**
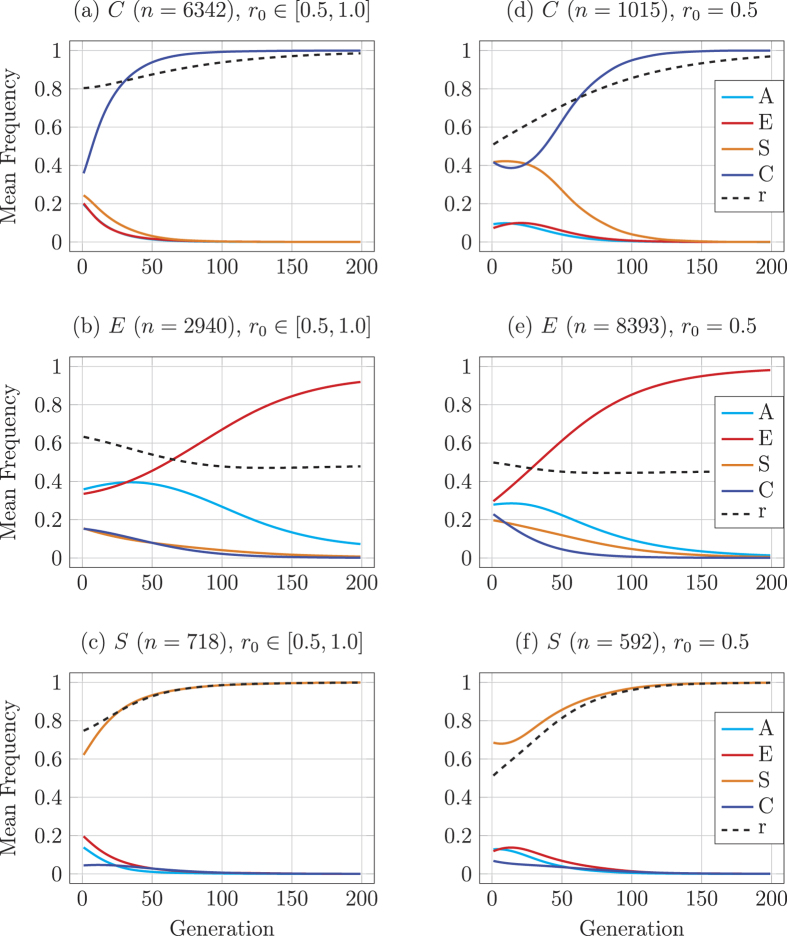
Mean evolutionary trajectories for the simulations of discerning recognition. Runs that converge on the three dynamically stable equilibria *C, E*, or *S* are represented separately where *n* is the number of runs (out of 10000) that converge on each equilibrium and *r*_0_ is the initial value of recognition ability *r*. The left column (**a**–**c**) gives mean trajectories for simulation results when *r*_0_ is drawn from an uniform distribution over [0.5, 1.0]; the right column (**d**–**f**) gives results for *r*_0_ = 0.5. Simulations run with *p* = 0.4, for equal values for help conferred or harm inflicted (*b* = *h* = 1), equal costs to confer help or inflict harm (*c*_*h*_ = *c*_*b*_ = 0.2), with mutation (*μ* = 0.0001).

Figure 3 describes the mean populations frequencies over time for each subset of simulation runs that converge on a specific stable equilibrium point (near all *C, E*, or *S*). Recognition ability plays a crucial role in the evolution and stability of *C* and *S*. Figure 3a–c shows that populations which converge on those equilibria tend to start with high *r*, although *S* only evolves when *C* starts at a very low frequency and goes extinct quickly. Figure 3d–f compare the case where the system starts with no effective recognition ability (*r* = 0.5). In this case *C* can still evolve and promote recognition but the basin of attraction for the *C* equilibrium is significantly reduced.

**Figure 4:**
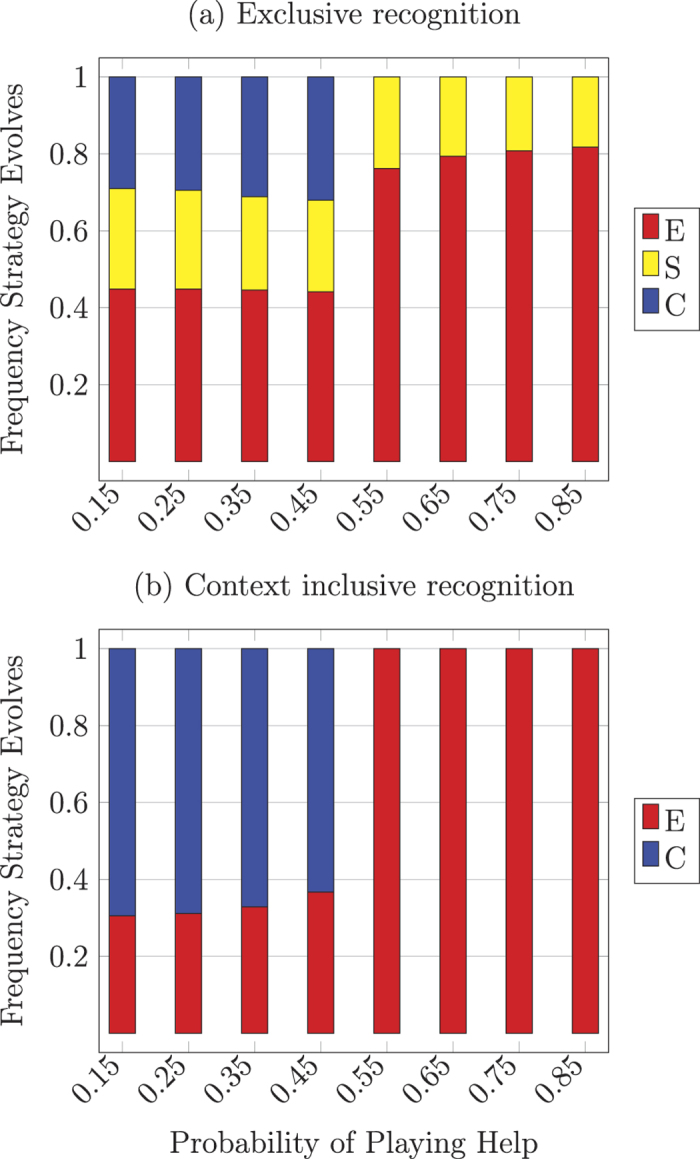
Simulation results for symmetric recognition: (**a**) exclusive and (**b**) context inclusive. All populations converge to a monomorphic equilibrium of one of the behavioral types *C, E*, or *S* in the exclusive case (*A* never evolves), and *C* or *E* in the context inclusive case (*A* or *S* never evolve). Simulation results show proportion of descendant population states from random initial conditions for equal values for help conferred or harm inflicted (*b* = *h* = 1), equal costs to confer help or inflict harm (*c*_*h*_ = *c*_*b*_ = 0.2), 10000 runs with mutation (*μ* = 0.0001).

The results are not driven by the asymmetries introduced by discerning recognition as the qualitative trend is robust with respect to symmetric exclusive and context inclusive recognition. Figure 4a shows the results for the model with exclusive recognition, where types consider only their identical type as similar. This kind of recognition increases the basin of attraction for the spiteful type *S* most dramatically. Figure 4b shows the results when recognition is context inclusive, where types only consider the type of interaction (e.g., *C* and *A* treat each other as similar in Help, but as different in Harm). Again, similar qualitative results hold with respect to the evolution of *C* and variation in *p*. However, *S* types always go extinct with context inclusive recognition. This is because *C* and *S* types treat each other as similar in Harm but *C* types only help fellow *C* types in Help. Since *S* types maintain high recognition, *C* can invade and take over *S* populations.

The coevolutionary model presented here and some of the results can be generalized to any two-player symmetric game (see Supplemental Information, Propositions 1–3). In particular, any conditional type, in any game, which attempts to enforce playing a dominated strategy by reverting to the dominant strategy will erode recognition. Additionally, types that adopt conditional behavior toward others which is both (i) harmful to potential invaders and (ii) costly to the natives can maintain high recognition ability. This suggest it is something like conditional spite (or some other external selection pressure), rather than altruism, which ultimately supports type recognition ability.

## Discussion

In our coevolutionary model conditional helping alone (*A*) cannot coevolve with recognition. Conditional helping can evolve if it is coupled with harmful behavior directed towards different types (as type *C* does), and Harm interactions occur sufficiently often (*C* is stable with respect to invasion by *E* when *p* ≤ *h*/(*h* + *c*_*b*_ − *b*) and *r* is maintained when *p* ≤ *c*_*h*_/(*c*_*h*_ + *c*_*b*_). Conditional harm (*S*) can coevolve with recognition only when the population starts with a sufficient proportion of *S* types.

These results have two major implications for the evolution of cooperation. First, they show that conditional altruism alone cannot maintain recognition. Any mechanism for the evolution of cooperation that presumes some sort of recognition ability requires that some additional evolutionary pressure be in place to maintain that recognition, otherwise cooperation will erode due to the covert free-riding permitted by recognition failure. One such evolutionary pressure that can maintain recognition is conditional harming behavior. Second, our coevolutionary model provides an alternative scenario for how cooperation can evolve. Conditional harming behavior and correspondingly high recognition ability can evolve first. Studies on the evolution of spite show that such harmful behavior can evolve in a range of conditions^12^,^24^,^25^. Once high *r* evolves then cooperative strategies can readily invade and evolve. So long as the conditional harming behavior maintains high *r*, robust cooperative behavior can be stable. This contrasts with the usual story about the evolution of punishment^8^,^21^,^22^. Here we have shown how it is possible for the conditional harming behavior we tend to associate with punishment to evolve before any cooperative norms or behaviors existed for such harm to enforce.

To reinforce this point, consider a possible way for the combination strategy *C* to originate in the current model. While our coevolutionary model does not address the origin of spite, it can originate in a number of ways without recognition^12^,^24^,^25^. Our model shows that if initial frequencies are sufficiently high, conditional spite will coevolve with recognition. A population of *S* types always playing Harm will maintain a high level of recognition and be stable (though such a population would be neutrally stable with respect to *C* types if recognition is context inclusive). If rare opportunities to Help begin to arise, and certain *S* type individuals are predisposed to help each other in these interactions, this effectively invents the *C* type. If this new type uses context inclusive recognition, then *C* can invade and fix in the population due to a combination of the high recognition maintained by *S* types and the benefits of cooperation in the rare opportunities to play Help. The *C* type is less likely to originate from *A* due to the instability of *A* types and recognition.

Our model does not presume a particular mechanism of recognition and so applies broadly to cases where recognition may be mediated by kin relationships, greenbeard genes, or other markers. Instantiating the mechanism of recognition in different ways can produce different models with more complex dynamical behavior. One such study examined the coevolution of neutral markers and marker-based conditional helping and harming in finite panmictic and infinite structured populations^15^. This study generalizes the marker-based recognition familiar from greenbeard models^28^, which build in an association between the marker (a green beard) and the conditional behavior (help those with green beards). Our model differs from both approaches in that we treat recognition as a continuous trait without any specific genetic linkage to social behavior. This allows us to examine varying degrees of imperfect conditional behavior rather than perfect conditional behavior that is neutral-marker specific, and whether the association between recognition and social behavior can emerge from the coevolutionary system. Since failures of recognition permit covert free-riding in Help interactions, conditional helping can evolve and be stable only when high recognition can be maintained. This can be achieved by coupling conditional help with conditional harm (e.g., the *C* types in this model), or by maintaining the linkage through genetic or demographic means. Thus, the results here support the previous finding^15^ that conditional harming evolves under a wider range of demographic conditions than conditional helping.

Another way to recognize an individual’s behavioral type is to observe past behavior in repeated interactions. Both direct and indirect reciprocity can facilitate the evolution of cooperation^2^,^3^,^9^. One study argues that the evolution of reciprocal cooperation is unlikely because it requires the simultaneous evolution of both the ability to cooperate and the the ability to respond conditionally, and neither ability would be favored in the absence of the other^31^. While our model does not directly address recognition mediated by past interactions with the same individuals, as is the case with repeated games, it does illustrate another potential difficulty for conditional behavior: insofar as the conditional behavior in repeated games is mediated by a signal or cue we should expect it to erode in cooperative contexts. Context inclusive recognition could be mediated by observations of past behaviors; observing past helping and harming behaviors would signal that the individual is a similar type. However, the model would need to be extended, for if recognition erodes then similar types help less frequently than they free ride. Such an extension to recognition and social behavior in repeated interactions is a promising direction for future research.

The generality of the coevolutionary model permits other applications as well. For example, the model may help us understand the evolutionary dynamics surrounding immune systems. These systems benefit from high degrees of recognition and direct conditionally harmful responses. The immune responses need to be directed at pathogens and foreign elements, not at endogenous cells or tissues. Failures of recognition can result in pathogenic infection (when foreign pathogens go unrecognized) or auto-immune disorders (when the immune system attacks endogenous cells), and we should expect to see a spectrum of mechanisms and responses to mediate recognition^32^. One evolutionary possibility is that the high recognition facilitated by immune systems may help contribute to cooperation and division of labor among endogenous cell lineages, an important concern for broad evolutionary hypotheses about major transitions and the evolution of individuality^33^.

## Methods

### Fitness Functions

Let *x* = (…, *x*_*i*_, …) represent the distribution of types in the population where *x*_*i*_ is the frequency of type *i*. Let *r* ∈ [0, 1] represent the mean recognition ability in the population and *r*′ be the recognition of the actor type. Evolutionary change will occur simultaneously on the distribution of types and on the recognition ability.

Types are defined by specified conditional strategies. The payoffs between types are determined by the conditional strategies, the recognition ability, and specifying the kind of recognition. More precisely, we define a function *u*_*g*_(*i, j, r*′, *r*) which determines the average payoff for type *i* against type *j* in game *g* when *i* has recognition ability *r*′ and *j* has recognition ability *r*. For the specific model considered here, where individuals play Help with probability p, Harm with probability (1−p) and there are four types: *A, E, S*, and *C*, we can express the utility function using a set of matrices for each type of recognition: *discerning* (Table 1), *exclusive* (Table 2), and *context inclusive* (Table 3). A general method for specifying *u*_*g*_(*i, j, r*′, *r*) is given in the Supplemental Information.

To determine the fitness of behavioral types we assume all individuals have the same recognition ability as the population mean (i.e. *r*′ = *r*). Let *F*(*i, x, r*) be the fitness of type *i* in population *x* with mean recognition ability *r*. For the combined Bayesian game, where Help is played with probability *p* and Harm with probability (1 − *p*), the average payoff of type *i* becomes a convex combination of the payoffs in Help and Harm. When individuals interact randomly and play the combined Bayesian game in an infinite population then


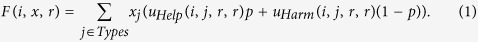


The average fitness of the population is


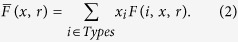


To consider the fitness of alternative recognition values *r*′, let 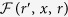 denote the fitness of a recognition ability *r*′ in a population *x* with mean recognition ability *r*:


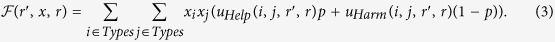


An alternative recognition ability *r*′ will be favored by selection whenever 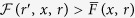. Note that because 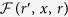 is linear with respect to *r*′, any *r*′ > *r* is such that 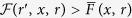 if and only if 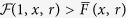. This allows us to use the simpler 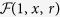 to determine evolutionary trends (see Supplemental Information).

### Stability

We can consider the evolutionary stability of types and their ability to maintain recognition. The most interesting case is that of type *C*. Assuming perfect recognition (*r* ≈ 1), the relevant inequality for the strong evolutionary stability of *C* with respect to types is *F*(*C, x, r*) > *F*(*E, x, r*) when *x*_*c*_ ≈ 1, this is approximated by:


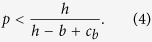


This inequality holds for all three cases of recognition. Types *A* and *S* are less of a threat to type *C*. Furthermore, if *h* and *b* are similar in magnitude, this condition should be easily satisfied provided a relatively small cost *c*_*b*_.

The key condition for the stability of type *C* in this model is the condition for maintaining high recognition. The condition for maintaining recognition for a population of *C* types is 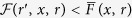 for *r*′ < *r*. For *x*_*c*_ ≈ 1 and *r* ≈ 1 this condition is approximated by the following inequality:


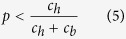


The relative *costs*, not the magnitudes of the harm and benefit, determine of whether the population will maintain a high degree of recognition. This inequality also illustrates why the transition point for the stability of *C* types relative to *E* is at *p* = 0.5 when *c*_*h*_ = *c*_*b*_. If *c*_*h*_ > *c*_*b*_ then the threshold will be *p* > 0.5 and if *c*_*b*_ > *c*_*h*_ then the threshold will be *p* < 0.5. In fact, relative to preventing invasion from *E*, this same inequality holds for types *A* and *S* as well, but with *c*_*h*_ = 0 for the former and *c*_*b*_ = 0 for the latter. Hence, the inequality cannot be satisfied for any positive *p* value with respect to *A* and is trivially satisfied in the case of *S*. This does not mean, however, that *A* and *S* will always be stable as the former will always be destabilized by deterioration in recognition ability and the latter can be destabilized by *C* if recognition is context-inclusive.

### Evolutionary Dynamics

The discrete-time replicator dynamics are given by the following difference equations where 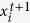 represents the frequency of type *i* in the next generation and *μ* is the mutation rate:


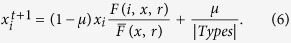


We assume that mutation is equally likely to occur among all types.

To represent the dynamic evolution of the recognition ability, we introduce an additional discrete-time dynamic that operates on *r* as though perfect (*r*′ = 1) and mean recognition (*r*) attempts were “types” in the population. Recognition ability increases proportionally to the increase of fitness from perfect recognition:


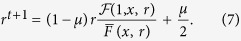


The recognition ability and its evolution are not directly tied to or connected with behavioral types. We assume the evolutionary dynamics operate independently connected only through the fitness calculations. Correlations between conditional behavior and recognition can thus emerge from the coevolutionary dynamics. Additionally, the dynamics can also be extended to include variation in the relative rates of evolution by introducing lower variation in the *r* population. These variations on the model produce similar qualitative results (see Supplemental Information).

For numerical simulations we initialize a population by drawing from an uniform distribution over the simplex of types and from an uniform distribution over the interval [0.5, 1] for recognition ability (for one set of simulations we set the initial recognition ability to *r* = 0.5; see Fig. 3d–f). In each generation the fitnesses for types are calculated using the current value of *r*, and the fitnesses for perfect versus mean recognition are calculated based on the current distribution of types *x*. Then both the type population *x* and the recognition ability *r* are updated according to the dynamics described. Unless otherwise specified in the results, simulations were run for 10^6^ generations.

## Additional Information

**How to cite this article**: Smead, R. and Forber, P. The coevolution of recognition and social behavior. *Sci. Rep.* **6**, 25813; doi: 10.1038/srep25813 (2016).

## Supplementary Material

Supplementary Information

## Figures and Tables

**Table 1:** Type payoffs for Help (top) and Harm (bottom) with discerning recognition.

	A	E	S	C
A	*br* − *c*_*b*_*r*′	−*c*_*b*_ (1 − *r*′)	−*c*_*b*_ (1 − *r*′)	*b* (1 − *r*) − *c*_*b*_*r*′
E	*b* (1 − *r*)	0	0	*b* (1 − *r*)
S	*b* (1 − *r*)	0	0	*b* (1 − *r*)
C	*br* − *c*_*b*_ (1 − *r*′)	−*c*_*b*_ (1 − *r*′)	−*c*_*b*_ (1 − *r*′)	*br* − *c*_*b*_*r*′
	**A**	**E**	**S**	**C**
A	0	0	−*hr*	−*hr*
E	0	0	−*hr*	−*hr*
S	−*c*_*h*_*r*′	−*c*_*h*_*r*′	−*h* (1 − *r*) − *c*_*h*_ (1 − *r*′)	−*hr* − *c*_*h*_ (1 − *r*′)
C	−*c*_*h*_*r*′	−*c*_*h*_*r*′	−*h* (1 − *r*) − *c*_*h*_*r*′	−*h* (1 − *r*) − *c*_*h*_ (1 − *r*′)

**Table 2:** Type payoffs for Help (top) and Harm (bottom) with exclusive recognition.

	A	E	S	C
A	*br* − *c*_*b*_*r*′	−*c*_*b*_ (1 − *r*′)	−*c*_*b*_ (1 − *r*′)	*b* (1 − *r*) − *c*_*b*_ (1 − *r*′)
E	*b* (1 − *r*)	0	0	*b* (1 − *r*)
S	*b* (1 − *r*)	0	0	*b* (1 − *r*)
C	*b* (1 − *r*) − *c*_*b*_ (1 − *r*′)	−*c*_*b*_ (1 − *r*′)	−*c*_*b*_ (1 − *r*′)	*br* − *c*_*b*_*r*′
	**A**	**E**	**S**	**C**
A	0	0	−*hr*	−*hr*
E	0	0	−*hr*	−*hr*
S	−*c*_*h*_*r*′	−*c*_*h*_*r*′	−*h* (1 − *r*) − *c*_*h*_ (1 − *r*′)	−*hr* − *c*_*h*_*r*′
C	−*c*_*h*_*r*′	−*c*_*h*_*r*′	−*hr* − *c*_*h*_*r*′	−*h* (1 − *r*) − *c*_*h*_ (1 − *r*′)

**Table 3:** Type payoffs for Help (top) and Harm (bottom) with context inclusive recognition.

	A	E	S	C
A	*br* − *c*_*b*_*r*′	−*c*_*b*_ (1 − *r*′)	−*c*_*b*_ (1 − *r*′)	*br* − *c*_*b*_*r*′
E	*b* (1 − *r*)	0	0	*b* (1 − *r*)
S	*b* (1 − *r*)	0	0	*b* (1 − *r*)
C	*br* − *c*_*v*_*r*′	−*c*_*b*_ (1 − *r*′)	−*c*_*b*_ (1 − *r*′)	*br* − *c*_*b*_*r*′
	**A**	**E**	**S**	**C**
A	0	0	−*hr*	−*hr*
E	0	0	−*hr*	−*hr*
S	−*c*_*h*_*r*′	−*c*_*h*_*r*′	−*h* (1 − *r*) − *c*_*h*_ (1 − *r*′)	−*h* (1 − *r*) − *c*_*h*_ (1 − *r*′)
C	−*c*_*h*_*r*′	−*c*_*h*_*r*′	−*h* (1 − *r*) − *c*_*h*_ (1 − *r*′)	−*h* (1 − *r*) − *c*_*h*_ (1 − *r*′)
