# Supplementary material for: The coevolution of recognition and social behavior
Source: Sci Rep. 2016 May 26;6:25813. doi: 10.1038/srep25813 (PMC4881042; doi:10.1038/srep25813)
Supplement: Supplementary Information [file srep25813-s1.pdf]

The coevolution of recognition and social behavior

Rory Smead[1] and Patrick Forber[2]

May 4, 2016

Affiliations

[1] (Corresponding author) Department of Philosophy and Religion, Northeastern University, Holmes Hall, 360 Huntington Ave, Boston, MA 02115 USA, r.smead@neu.edu

[2] Department of Philosophy, Tufts University, Miner Hall, 14 Upper Campus Rd, Medford, MA 02155 USA, patrick.forber@tufts.edu

Supplemental Information

Here we present a model that generalizes the approach used in the main text to any two player symmetric game. We show that the results regarding the evolutionary instability of conditional helping and the evolutionary stability of conditional harming are general. We consider both the stability of behavioral types in any two player symmetric game as well as the stability of recognition in monomorphic populations. Finally, we present an alternative selection dynamics on recognition which alters the relative rate of evolution, but produces qualitatively similar results to those presented in the main text.

A generalized model

For any two player symmetric game, let $\pi(\sigma, \sigma')$ be the payoff of behavior σ played against σ'. As we are considering conditional strategies in these games, we will refer to choices within the game as "behaviors" and the full conditional strategies (e.g., do behavior x against similar types and behavior y against different types) as "types." Let $i \rightharpoonup j$ denote that i considers j a similar type (but j does not consider i similar) and $i \rightleftharpoons j$ denote that both types consider each other similar. As in the main text, r represents recognition ability. Let s_i be the behavior that type i adopts against similar types and d_i be the behavior they adopt against different types. The generalized utility function can be specified as follows. When $i \rightleftharpoons j$:

$$\begin{aligned} u(i, j, r', r) = {} & r'r\pi(s_i, s_j) + r'(1-r)\pi(s_i, d_j) \\ & + (1-r')r\pi(d_i, s_j) + (1-r')(1-r)\pi(d_i, d_j). \end{aligned} \tag{1}$$

Where r' is the recognition ability of i and r is the recognition ability of j. For $i \rightharpoonup j$ simply swap s_j and d_j in Equation 1. Similarly for $i \leftharpoondown j$, swap s_i and d_i in Equation 1. For the case where neither type considers the other type similar, swap s_i and d_i as well as s_j and d_j throughout. Finally, we will use $u(i, j, r)$ to denote the case where both players have the

same recognition value ($r' = r$).

A population state $x = (..., x_i, ...)$ is a point in the unit simplex which represents the frequency of each type i with a value $x_i \in [0, 1]$. Let $F(i, x, r)$ be the fitness of type i in population x with a mean recognition ability r. When recognition ability is uncorrelated with type and the population is infinitely large this is

$$F(i, x, r) = \sum_{j \in Types} x_j u(i, j, r). \tag{2}$$

The average fitness in the population is

$$\overline{F}(x, r) = \sum_{i \in Types} x_i F(i, x, r). \tag{3}$$

It is also possible to determine the fitness of a population state relative to another population state by treating those states as a mixed type (a mixed strategy in the type game) equivalent to the frequency of types in the population [1]. The fitness of population x' playing against population x is given by $F(x', x, r)$ according to equation 2 where a weighted average of types in x' is used in place of i: $F(x', x, r) = \sum_i \sum_j x'_i x_j u(i, j, r)$. This will be helpful in thinking about evolutionary stability in the next section.

Finally, to consider the fitness of alternative recognition values r' relative to a population mean r, we use the full utility function $u(i, j, r', r)$. Let $\mathcal{F}(r', x, r)$ denote the fitness of a recognition ability r' in a population x with mean recognition ability r:

$$\mathcal{F}(r', x, r) = \sum_{i \in Types} \sum_{j \in Types} x_i x_j u(i, j, r', r). \tag{4}$$

Recognition ability r' will be favored by selection whenever $\mathcal{F}(r', x, r) > \overline{F}(x, r)$. Because $u(i, j, r', r)$ is linear with respect to r', we can determine whether or not an alternative $r' > r$ is favored by selection by simply examining the case of a perfectly accurate recognizer

$\mathcal{F}(1, x, r)$ in comparison with the mean fitness of the population: $r' > r$ will be favored whenever $\mathcal{F}(1, x, r) > \overline{F}(x, r)$. Likewise, $r' < r$ will be favored if and only if $\mathcal{F}(0, x, r) < \overline{F}(x, r)$.

Stability of types

Standard fitness comparisons (with mean fitness) can be used to determine whether types will increase in frequency according to any monotonic selection dynamic. Likewise, given a fixed recognition value, we can define stability with respect to types in a manner similar to the standard definition of an Evolutionarily Stable Strategy [1, 2].

Definition 1. *A population state* (x, r) *is* evolutionarily stable with respect to types *if and only if (i)* $F(x, x, r) > F(x', x, r)$ *or (ii)* $F(x, x, r) = F(x', x, r)$ *and* $F(x, x', r) > F(x', x', r)$ *for all* $x' \neq x$*.*

If a population state satisfies the first condition, we will call it *strongly* evolutionarily stable.

Before considering evolutionary stability with respect to recognition, it is possible to demonstrate some necessary and sufficient conditions regarding stability with respect to types in populations with high recognition.

Propositon 1. *If* (x, r) *is such that* $x_i \approx 1$ *and* $r \approx 1$*, then for* (x, r) *to be evolutionarily stable with respect to types, it is necessary that* $\pi(s_i, s_i) \geq \pi(s, d_i)$ *for all behaviors* s*.*

Proof. Suppose that $x_i \approx 1$, $r \approx 1$ and $\pi(s_i, s_i) < \pi(s, d_i)$ for some behavior s. Note that we cannot have $s = d_i = s_i$. Let j be such that $d_j = s$. In which case, $\pi(s_i, s_i) < \pi(j, d_i)$ and, because $r \approx 1$, $u(i, i, r) < u(j, i, r)$. Lastly, if $x_i \approx 1$, $F(x, x, r) < F(j, x, r)$. Thus, (x, r) is not evolutionarily stable with respect to types. □

In words, this proposition shows that when recognition is high no monomorphic population can be stable if native's conditional behavior d_i benefits some alternative behavior that a

different type may choose. If there is such a behavior, then any type which adopts that behavior against type i will have a strict fitness advantage over the natives. The next proposition shows that if the inequality in Proposition 1 is strict, it is a sufficient condition for evolutionary stability with respect to types.

Propositon 2. *If (x, r) is such that $x_i \approx 1$ and $r \approx 1$, then for (x, r) to be evolutionarily stable with respect to types, it is sufficient that $\pi(s_i, s_i) > \pi(s, d_i)$ for all behaviors s.*

Proof. Suppose $x_i \approx 1$, $r \approx 1$ and that $\pi(s_i, s_i) > \pi(s, d_i)$ for all behaviors s. Then, for every type $j \neq i$, $\pi(s_i, s_i) > \pi(d_j, d_i)$. Thus, $u(i, i, r) > u(j, i, r)$ and, since $x_i \approx 1$, $F(x, x, r) > F(x', x, r)$ for all x' nearby x and (x, r) is strongly evolutionarily stable with respect to types. □

Proposition 2 shows that any conditional behavior that effectively *harms* alternative types will be evolutionarily stable. This is harm in a relative sense: alternative types against the natives receive a payoff less than that of the average in the native population.

Stability of recognition ability

We are specifically interested in populations that can maintain high recognition and will demonstrate the conditions under which higher values of recognition are favored over lower values in monomorphic populations. The single dimension of the r parameter and the fact that $u(i, j, r', r)$ is linear with respect to r' allows a straightforward definition.

Definition 2. *A population state (x, r)* favors recognition *if and only if (i) $\mathcal{F}(r', x, r) > \overline{F}(x, r)$ for all $r' > r$ and (ii) $\mathcal{F}(r', x, r) < \overline{F}(x, r)$ for all $r' < r$.*

To determine whether or not a monomorphic population can favor high recognition we need to compare cases of successful recognition to those of unsuccessful recognition. Because we are assuming two player symmetric games, this comparison can be done by considering

	s_i	d_i
s_i	a	b
d_i	c	d

Table 1: The sub-game between s_i and d_i for a specific type i.

the behavior used when the native type i makes a "similar" determination (s_i) against others of that type and the behavior used when i makes a "different" determination (d_i) against other type i players. The interaction between these component behaviors s_i and d_i can be summarized in Table 1. Note that a similar matrix has been used to represent the space of all possible 2×2 interactions [3]. Also note that Table 1 describes only one part of a potentially much larger game. However, in a monomorphic population s_i and d_i are the only strategies that will be realized with measurable frequency. With this interaction component we are able to express general conditions for when recognition will be favored in monomorphic populations.

Propositon 3. *Recognition is favored in a monomoprhic population if and only if* $r(a - c) > (1 - r)(d - b)$.

Proof. Supppose $x_i \approx 1$. Recognition will be favored whenever (i) $\mathcal{F}(r', x, r) > \overline{F}(x, r)$ for all $r' > r$ and (ii) $\mathcal{F}(r', x, r) < \overline{F}(x, r)$ for all $r' < r$. First consider $r' > r$. Because $u(i, j, r', r)$ is linear with respect to r' and $x_i \approx 1$:

$$\begin{aligned}
\mathcal{F}(r', x, r) > \overline{F}(x, r) &\text{ iff } \mathcal{F}(1, x, r) > \overline{F}(x, r) \\
&\text{ iff } u(i, i, 1, r) > u(i, i, r) \\
&\text{ iff } ra + (1 - r)b > r^2 a + r(1 - r)b + (1 - r)rc + (1 - r)^2 d \\
&\text{ iff } r(a - c) > (1 - r)(d - b)
\end{aligned}$$

Next consider $r' < r$:

$$\begin{aligned}\mathcal{F}(r', x, r) < \overline{F}(x, r) &\text{ iff } \mathcal{F}(0, x, r) < \overline{F}(x, r)\\ &\text{ iff } u(i, i, 0, r) < u(i, i, r)\\ &\text{ iff } rc + (1-r)d < r^2 a + r(1-r)b + (1-r)rc + (1-r)^2 d\\ &\text{ iff } r(a-c) > (1-r)(d-b)\end{aligned}$$

Therefore, recognition is favored in (x, r) if and only if $r(a-c) > (1-r)(d-b)$.

□

Proposition 3 shows that there are only certain types that can favor recognition ability. This can apply readily to important classes of games. For example, consider a conditionally altruistic type in the Prisoner's Dilemma, which cooperates with similar types and defects on different types (i.e., type A in the Help Game). In this case, we have $c > a > b > d$ and consequently conditional altruism can never maintain recognition on its own. Proposition 3 shows that this will be a general trend in any game with a conditional type attempting to maintain a dominated strategy in a similar way—i.e., where the conditional type plays the dominated strategy against its own type and the dominant strategy against others.

Additionally, for any monomorphic population to maintain a high degree of recognition ($r \approx 1$) it becomes essential that $a > c$. This means that that it must be, in a relative sense, *costly* for the native population to execute their different-targeted conditional behavior against other natives. Combining this result from that of Proposition 2 shows that conditional spite (costly behavior that inflicts harm) is sufficient to stabilize both type and recognition (e.g. type S in the Harm game). This is not to say that games such as the Harm game are the only interactions that can sustain high recognition values. Other games may provide the requisite settings as well, provided that the conditional behavior d_i is sufficiently harmful to potential invaders and sufficiently costly to the natives.

Variance of recognition and speed of selection

In the dynamic model we implemented a discrete-time selection dynamic on recognition:

$$r^{t+1} = (1-\mu)r\frac{\mathcal{F}(1,x,r)}{\overline{F}(x,r)} + \frac{\mu}{2}. \tag{5}$$

This dynamic considers only *perfect* recognition in comparison with mean fitness. While we saw above that this is sufficient to draw conclusions about the direction of selection (higher or lower r'), it also assumes that selection will operate at a very fast speed, as though the population had the highest possible variance among r values. To account for the possibility of lower variation in recognition ability relative to that of behavioral dispositions, we introduce a variance term into the evolutionary dynamics that are operating on r. Let $r_{var} \in [0,1]$ be a variance parameter within the population with respect to recognition ability and r_Δ represent the difference between the fitness of successful recognition and the mean fitness:

$$r_\Delta = \mathcal{F}(1,x,r) - \overline{F}(x,r). \tag{6}$$

Then we can modify equation 5 to slow evolution in proportion to the degree of variance in recognition ability. This is a linear transformation of the fitness that effectively allows us to consider deviations of recognition value that are much closer to the mean value of the population:

$$r^{t+1} = (1-\mu)r\frac{\mathcal{F}(1,x,r) - (1-r_{var})r_\Delta}{\sum_i x_i F(i,x,r)} + \frac{\mu}{2}. \tag{7}$$

We ran an additional set of simulations using the dynamics expressed in Equation 7. These simulations produced results that were qualitatively similar to the results of the main text. These results show that using a perfect-recognition as the comparison point for the evolutionary dynamics does not change the qualitative results of the model. See Figure 1.

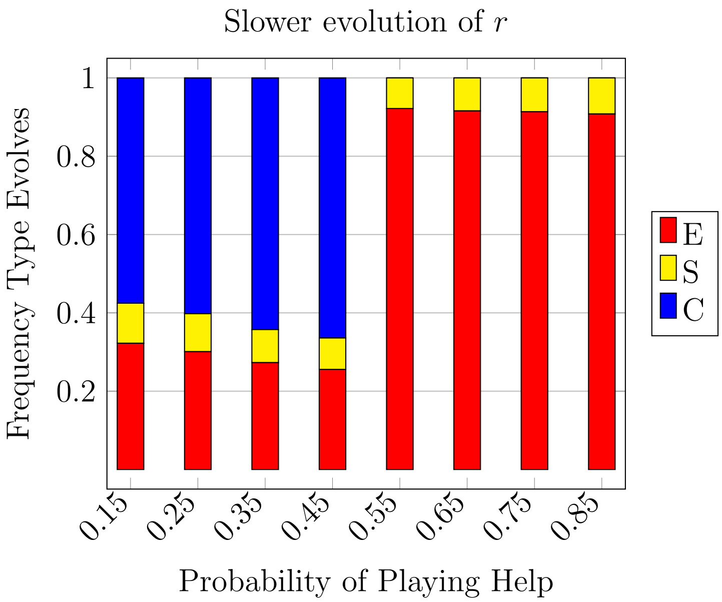

Figure 1: Simulation results for slower evolution of r. All populations converge to a monomorphic equilibrium of one of the behavioral types E, S, or C (A never evolves). Simulation results show proportion of descendant population states from random initial conditions for $rvar = 0.1$, equal values for help conferred or harm inflicted ($b = h = 1$), equal costs to confer help or inflict harm ($c_h = c_b = 0.2$), 10000 runs with mutation ($\mu = 0.0001$).

References

[1] Sandholm, W. H. *Population Games and Evolutionary Dynamics* (MIT Press, 2010).

[2] Maynard Smith, J. & Price, G. R. The logic of animal conflict. *Nature* **246**, 15–18 (1973).

[3] Weibull, J. W. *Evolutionary Game Theory* (MIT Press, 1995).
